# High-quality permanent draft genome sequence of *Bradyrhizobium* sp. Th.b2, a microsymbiont of *Amphicarpaea bracteata* collected in Johnson City, New York

**DOI:** 10.1186/s40793-015-0008-y

**Published:** 2015-05-16

**Authors:** Rui Tian, Matthew Parker, Rekha Seshadri, TBK Reddy, Victor Markowitz, Natalia Ivanova, Amrita Pati, Tanja Woyke, Mohammed N Baeshen, Nabih A Baeshen, Nikos Kyrpides, Wayne Reeve

**Affiliations:** 1 Centre for Rhizobium Studies, Murdoch University, Murdoch, Australia; 2 Binghamton University, State University of New York, New York, USA; 3 DOE Joint Genome Institute, Walnut Creek, California, USA; 4 Biological Data Management and Technology Center, Lawrence Berkeley National Laboratory, Berkeley, California, USA; 5 Department of Biological Sciences, Faculty of Science, King Abdulaziz University, Jeddah, Saudi Arabia; 6 Center of Nanotechnology, King Abdulaziz University, Jeddah, Saudi Arabia; 7 Department of Biological Sciences, Faculty of Science, Jeddah University, Jeddah, Saudi Arabia

**Keywords:** Root-nodule bacteria, Nitrogen fixation, Symbiosis, Alphaproteobacteria, GEBA-RNB

## Abstract

*Bradyrhizobium* sp. Th.b2 is an aerobic, motile, Gram-negative, non-spore-forming rod that was isolated from an effective nitrogen-fixing root nodule of *Amphicarpaea bracteata* collected in Johnson City, New York. Here we describe the features of *Bradyrhizobium* sp. Th.b2, together with high-quality permanent draft genome sequence information and annotation. The 10,118,060 high-quality draft genome is arranged in 266 scaffolds of 274 contigs, contains 9,809 protein-coding genes and 108 RNA-only encoding genes. This rhizobial genome was sequenced as part of the DOE Joint Genome Institute 2010 Genomic Encyclopedia for Bacteria and Archaea-Root Nodule Bacteria (GEBA-RNB) project.

## Introduction

Strain Th.b2 is a representative of a widely distributed *Bradyrhizobium * lineage used by several common legumes indigenous to forested habitats in eastern North America. Strain Th.b2 was sampled in 1991 from a population of the annual legume Amphicarpaea bracteata in Johnson City, NY. Surveys of other *A. bracteata* populations in the eastern United States based on 20 isozyme markers found that strains similar or identical to Th.b2 were present in 19 of 24 sites across six states (IL, IN, WI, MI, NY, PA [1]). Based on both isozyme data and rRNA sequencing, isolates that were similar or identical to Th.b2 were also detected in nodule samples from two common herbaceous perennial legumes, Apios americana and Hylodesmum glutinosum, that often occur in woodland habitats together with Amphicarpaea bracteata[2]*.* A multilocus sequence analysis found strains in North Carolina populations of *A. bracteata* that were similar or identical to Th.b2 [3], and also detected a highly similar strain on another herbaceous perennial legume, Desmodium paniculatum, that is widely distributed across eastern North America [4].

Based on these field surveys, the *Bradyrhizobium * lineage represented by strain Th.b2 appears to be relatively host-specific to legumes in these four genera (Amphicarpaea*,*Apios*,*Desmodium*, Hylodesmum*), because widespread sampling of sympatric legumes in eleven other genera have not detected this group [3],[5],[6]. However, inoculation experiments are needed to understand whether the Th.b2 lineage lacks the ability to nodulate these other genera, or alternatively, may simply be a poor competitor for nodulation in the presence of other bacterial strains that are their preferred symbionts.

It should also be noted that the eastern North American symbionts of Amphicarpaea*,*Apios*, Desmodium* and *Hylodesmum* are not phylogenetically homogeneous at housekeeping loci. Horizontal transfer of the symbiosis island (SI) region of the *Bradyrhizobium * chromosome [7] from a member of the Th.b2 clade to a distantly related *Bradyrhizobum* lineage has apparently enabled the recipient to gain the ability to interact with some of the normal legume hosts of the Th.b2 clade [3].

Bacteria that are closely related to Th.b2 have also been found in Japan associated with an Asian species of Amphicarpaea (*A. edgeworthii*) [6]. Surprisingly, strain Th.b2 lacks the ability to form nodules on *A. edgeworthii*, although Japanese strains from *A. edgeworthii* are effective nitrogen-fixing symbionts for the American legume *A. bracteata*[8],[9]. These differences appear to be related to variation between related East Asian and North American strains in the synthesis of rhizobitoxine [8].

Here we provide an analysis of the high-quality permanent draft genome sequence of *Bradyrhizobum * sp. Th.b2, one of the rhizobial genomes sequenced as part of the DOE Joint Genome Institute 2010 Genomic Encyclopedia for Bacteria and Archaea-Root Nodule Bacteria (GEBA-RNB) project proposal [10], whose properties may provide useful insights about the evolution of symbiotic specificity and its relationship to SI region horizontal transfer in *Bradyrhizobium *.

## Organism information

### Classification and features

**Figure 1:**
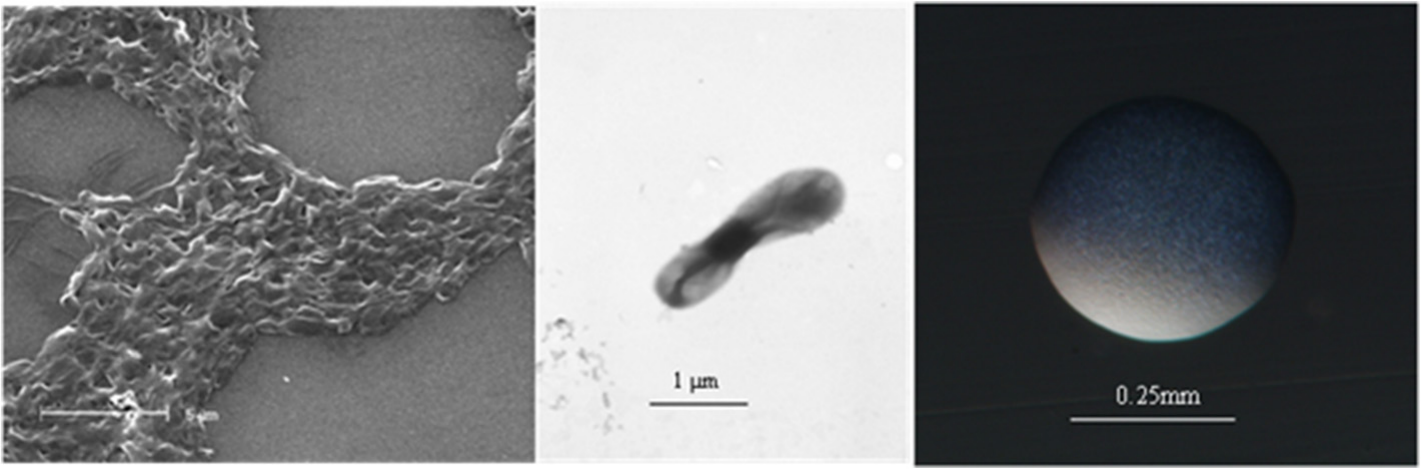
Images of *Bradyrhizobium* sp. Th.b2 using scanning (Left) and transmission (Center) electron microscopy as well as light microscopy to visualize colony morphology on solid media (Right).

*Bradyrhizobium * sp. Th.b2 is a motile, non-sporulating, non-encapsulated, Gram-negative strain in the order *Rhizobiales * of the class *Alphaproteobacteria *. The rod shaped form has dimensions of approximately 0.5 μm in width and 1.5-2.0 μm in length (Figure 1 Left and Center). It is relatively slow growing, forming colonies after 6–7 days when grown on half strength Lupin Agar (½LA) [11], tryptone-yeast extract agar (TY) [12] or a modified yeast-mannitol agar (YMA) [13] at 28°C. Colonies on ½LA are opaque, slightly domed and moderately mucoid with smooth margins (Figure 1 Right).

**Figure 2:**
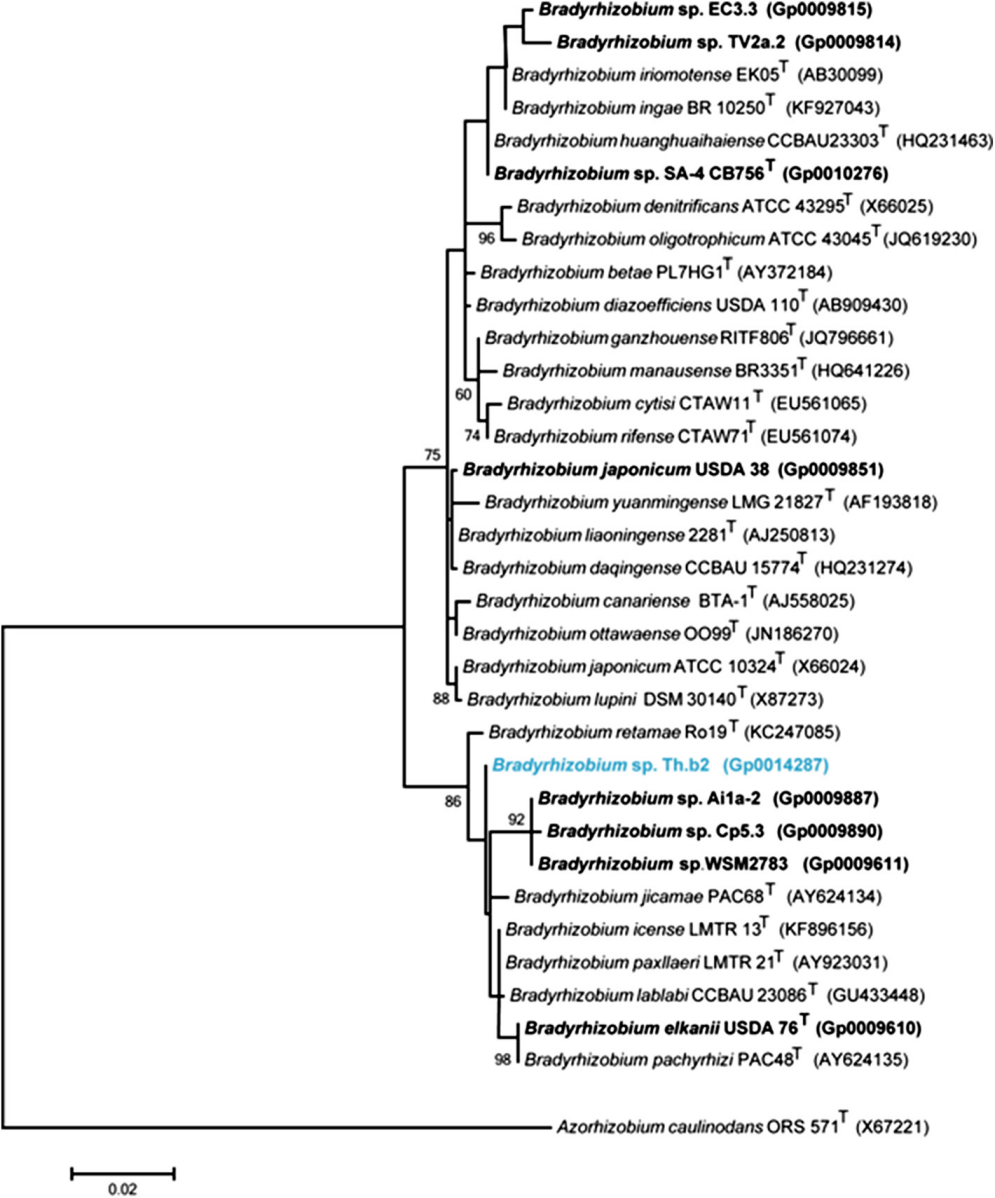
Phylogenetic tree highlighting the position of *Bradyrhizobium* sp. Th.b2 (shown in blue print) relative to other type and non-type strains in the *Bradyrhizobium* genus using a 1,310 bp intragenic sequence of the 16S rRNA gene. *Azorhizobium caulinodans* ORS 571^T^ sequence was used as an outgroup. All sites were informative and there were no gap-containing sites. Phylogenetic analyses were performed using MEGA, version 5.05 [21]. The tree was built using the maximum likelihood method with the General Time Reversible model. Bootstrap analysis with 500 replicates was performed to assess the support of the clusters. Type strains are indicated with a superscript T. Strains with a genome sequencing project registered in GOLD [16] have the GOLD ID mentioned after the strain number and are represented in bold, otherwise the NCBI accession number is provided.

Figure 2 shows the phylogenetic relationship of *Bradyrhizobium * sp. Th.b2 in a 16S rRNA gene sequence based tree. This strain is phylogenetically most closely related to the type strains *Bradyrhizobium icense * LMTR 13^T^ and *Bradyrhizobium paxllaeri * LMTR 21^T^, with a 16S rRNA gene sequence identity of 99.77% to the corresponding gene sequence of each type strain based on alignment using the EzTaxon-e server [14],[15].

Minimum Information about the Genome Sequence (MIGS) is provided in Table 1 and Additional file 1: Table S1.

**Table 1:** **Classification and general features of** ***Bradyrhizobium*** **sp. Th.b2 in accordance with the MIGS recommendations**[27]**published by the Genome Standards Consortium**[28]

**MIGS ID**	**Property**	**Term**	**Evidence code**
	Classification	Domain *Bacteria*	TAS [29]
		Phylum *Proteobacteria*	TAS [30],[31]
		Class *Alphaproteobacteria*	TAS [31],[32]
		Order *Rhizobiales*	TAS [33]
		Family *Bradyrhizobiaceae*	TAS [34]
		Genus *Bradyrhizobium*	TAS [35]
		Species *Bradyrhizobium* sp.	IDA
	Gram stain	Negative	IDA
	Cell shape	Rod	IDA
	Motility	Motile	IDA
	Sporulation	Non-sporulating	NAS
	Temperature range	Mesophile	NAS
	Optimum temperature	28°C	NAS
	pH range; Optimum	Unknown	NAS
	Carbon source	Varied	NAS
	Energy source	Chemoorganotroph	NAS
MIGS-6	Habitat	Soil, root nodule, host	TAS [1]
MIGS-6.3	Salinity	Non-halophile	NAS
MIGS-22	Oxygen requirement	Aerobic	NAS
MIGS-15	Biotic relationship	Free living, symbiotic	TAS [1]
MIGS-14	Pathogenicity	Non-pathogenic	NAS
	Biosafety level	1	TAS [36]
	Isolation	Root nodule of *Amphicarpaea bracteata*	TAS [1]
MIGS-4	Geographic location	Johnson City, New York	TAS [1]
MIGS-5	Sample collection date	1991	IDA
MIGS-4.1	Latitude	42.107	IDA
MIGS-4.2	Longitude	−75.9691	IDA
MIGS-4.3	Depth	5 cm	IDA
MIGS-4.4	Altitude	255 m	IDA

Evidence codes – IDA: Inferred from Direct Assay; TAS: Traceable Author Statement (i.e., a direct report exists in the literature); NAS: Non-traceable Author Statement (i.e., not directly observed for the living, isolated sample, but based on a generally accepted property for the species, or anecdotal evidence). Evidence codes are from the Gene Ontology project [37],[38].

### Symbiotaxonomy

Strain Th.b2 was isolated in 1991 from a population of the annual legume Amphicarpaea bracteata in Johnson City, NY. Isolates that were similar or identical to Th.b2 were also detected in nodule samples from two common herbaceous perennial legumes, Apios americana and Hylodesmum glutinosum, that often occur in woodland habitats together with Amphicarpaea bracteata[2]. Th.b2 lacks the ability to form nodules on the Asian species *Amphicarpaea. edgeworthii*, which is associated with a strain closely related to Th.b2 from Japan [6],[8].

## Genome sequencing information

### Genome project history

This organism was selected for sequencing on the basis of its environmental and agricultural relevance to issues in global carbon cycling, alternative energy production, and biogeochemical importance, and is part of the Genomic Encyclopedia of *Bacteria* and *Archaea*, Root Nodulating Bacteria (GEBA-RNB) project at the U.S. Department of Energy, Joint Genome Institute (JGI). The genome project is deposited in the Genomes OnLine Database [16] and a high-quality permanent draft genome sequence in IMG [17]. Sequencing, finishing and annotation were performed by the JGI using state of the art sequencing technology [18]. A summary of the project information is shown in Table 2.

**Table 2:** Project information

**MIGS ID**	**Property**	**Term**
MIGS-31	Finishing quality	High-quality permanent draft
MIGS-28	Libraries used	Illumina Standard PE
MIGS-29	Sequencing platforms	Illumina HiSeq2000
MIGS-31.2	Fold coverage	Illumina, 120.4x
MIGS-30	Assemblers	Velvet version 1.1.04; Allpaths-LG version r42328
MIGS-32	Gene calling method	Prodigal 1.4
	Locus Tag	K359
	GenBank ID	AUGA00000000
	GenBank Date of Release	June 13, 2014
	GOLD ID	Gp0014287 [46]
	BIOPROJECT	195826
MIGS-13	Source Material Identifier	Th.b2
	Project relevance	Symbiotic N_2_ fixation, agriculture

### Growth conditions and genomic DNA preparation

*Bradyrhizobium * sp. Th.b2 was cultured to mid logarithmic phase in 60 ml of TY rich media on a gyratory shaker at 28°C [19]. DNA was isolated from the cells using a CTAB (Cetyl trimethyl ammonium bromide) bacterial genomic DNA isolation method [20].

### Genome sequencing and assembly

The draft genome of *Bradyrhizobium * sp. th.b2 was generated at the DOE Joint Genome Institute (JGI) using the Illumina technology [22]. An Illumina standard shotgun library was constructed and sequenced using the Illumina HiSeq 2000 platform which generated 20,348,156 reads totaling 3,052.2 Mbp. All general aspects of library construction and sequencing were performed at the JGI and details can be found on the JGI website [23]. All raw Illumina sequence data was passed through DUK, a filtering program developed at JGI, which removes known Illumina sequencing and library preparation artifacts (Mingkun L, Copeland A, Han J, Unpublished). Following steps were then performed for assembly: (1) filtered Illumina reads were assembled using Velvet (version 1.1.04) [24], (2) 1–3 Kbp simulated paired end reads were created from Velvet contigs using wgsim [25], (3) Illumina reads were assembled with simulated read pairs using Allpaths–LG (version r42328) [26]. Parameters for assembly steps were: 1) Velvet (velveth: 63 –shortPaired and velvetg: −very clean yes –exportFiltered yes –min contig lgth 500 –scaffolding no –cov cutoff 10) 2) wgsim (−e 0 –1 100 –2 100 –r 0 –R 0 –X 0) 3) Allpaths–LG (PrepareAllpathsInputs: PHRED 64 = 1 PLOIDY = 1 FRAG_COVERAGE = 125 JUMP_COVERAGE = 25 LONG_JUMP_COV = 50, RunAllpathsLG: THREADS = 8 RUN = std_shredpairs TARGETS = standard VAPI_WARN_ONLY = True OVERWRITE = True). The final draft assembly contained 274 contigs in 266 scaffolds. The total size of the genome is 10.1 Mbp and the final assembly is based on 1,216.8 Mbp of Illumina data, which provides an average 120.4x coverage of the genome.

### Genome annotation

Genes were identified using Prodigal [39], as part of the DOE-JGI genome annotation pipeline [40],[41] The predicted CDSs were translated and used to search the National Center for Biotechnology Information (NCBI) non-redundant database, UniProt, TIGRFam, Pfam, KEGG, COG, and InterPro databases. The tRNAScanSE tool [42] was used to find tRNA genes, whereas ribosomal RNA genes were found by searches against models of the ribosomal RNA genes built from SILVA [43]. Other non–coding RNAs such as the RNA components of the protein secretion complex and the RNase P were identified by searching the genome for the corresponding Rfam profiles using INFERNAL [44]. Additional gene prediction analysis and manual functional annotation was performed within the Integrated Microbial Genomes-Expert Review (IMG-ER) system [45] developed by the Joint Genome Institute, Walnut Creek, CA, USA.

## Genome properties

The genome is 10,118,060 nucleotides with 63.25% GC content (Table 3) and comprised of 266 scaffolds. From a total of 9,919 genes, 9,809 were protein encoding and 108 RNA only encoding genes. The majority of genes (70.75%) were assigned a putative function whilst the remaining genes were annotated as hypothetical. The distribution of genes into COGs functional categories is presented in Table 4.

**Table 3:** **Genome statistics for** ***Bradyrhizobium*** **sp. Th.b2**

**Attribute**	**Value**	**% of Total**
Genome size (bp)	10,118,060	100.00
DNA coding (bp)	8,412,367	83.14
DNA G + C (bp)	6,399,174	63.25
DNA scaffolds	266	100
Total genes	9,917	100.00
Protein coding genes	9,809	98.91
RNA genes	108	1.09
Pseudo genes	0	0.00
Genes in internal clusters	713	7.19
Genes with function prediction	7,016	70.75
Genes assigned to COGs	5,576	56.23
Genes with Pfam domains	71.85	72.45
Genes with signal peptides	978	9.86
Genes coding transmembrane helices	2,166	21.84
CRISPR repeats	0	0.00

**Table 4:** Number of genes associated with the general COG functional categories

**Code**	**Value**	**% of total (6,228)**	**COG category**
J	199	3.20	Translation, ribosomal structure and biogenesis
A	0	0.00	RNA processing and modification
K	520	8.35	Transcription
L	197	3.16	Replication, recombination and repair
B	3	0.05	Chromatin structure and dynamics
D	30	0.48	Cell cycle control, cell division, chromosome partitioning
V	103	1.65	Defense mechanisms
T	248	3.98	Signal transduction mechanisms
M	290	4.66	Cell wall/membrane/envelope biogenesis
N	72	1.16	Cell motility
U	118	1.89	Intracellular trafficking, secretion, and vesicular transport
O	200	3.21	Posttranslational modification, protein turnover, chaperones
C	432	6.94	Energy production and conversion
G	382	6.13	Carbohydrate transport and metabolism
E	702	11.27	Amino acid transport and metabolism
F	81	1.30	Nucleotide transport and metabolism
H	208	3.34	Coenzyme transport and metabolism
I	391	6.28	Lipid transport and metabolism
P	338	5.43	Inorganic ion transport and metabolism
Q	301	4.83	Secondary metabolite biosynthesis, transport and catabolism
R	799	12.83	General function prediction only
S	614	9.86	Function unknown
-	4,341	43.77	Not in COGS

## Conclusions

*Bradyrhizobium * sp. Th.b2 was isolated from a root nodule of Amphicarpaea bracteata collected from Johnson City, New York. Little is currently known of the symbiotic associations of its host Amphicarpaea bracteata*.* This strain belongs to a member of a widely distributed *Bradyrhizobium * lineage, isolated from diverse legume hosts in North, Central and South America and South Africa. Phylogenetically, Th.b2 is separated from the most closely related species *Bradyrhizobium icense * LMTR 13^T^ and *Bradyrhizobium paxllaeri * LMTR 21^T^, both isolated from root nodules of Phaseolus lunatus (Lima bean) in Peru [47]. Th.b2 may therefore be a novel species of *Bradyrhizobium *. A total of 25 *Bradyrhizobium * genomes have now been sequenced as part of the GEBA-RNB project [10]. Of these 25 strains, Th.b2 has the second largest genome size (10.1 Mbp), gene count (9,917) and COG % and the lowest coding base count % (83.17). The genome attributes of *Bradyrhizobium * sp. Th.b2, in conjunction with other *Bradyrhizobium * genomes from GEBA-RNB project, will be important for the understanding of the biogeography of *Bradyrhizobium * spp. interactions required for the successful establishment of effective symbioses with their diverse hosts*.*

## Abbreviations

GEBA-RNB: Genomic Encyclopedia for Bacteria and Archaea-Root Nodule Bacteria

JGI: Joint Genome Institute

½LA: half strength Lupin Agar

TY: Tryptone Yeast

YMA: Yeast Mannitol Agar

CTAB: Cetyl Trimethyl Ammonium Bromide

## Competing interests

The authors declare that they have no competing interests.

## Authors’ contributions

MP supplied the strain and background information for this project and the DNA to the JGI, TR performed all imaging, TR and WR drafted the paper, MNB and NAB provided financial support and all other authors were involved in sequencing the genome and/or editing the final paper. All authors read and approved the final manuscript.

## Additional file

## Supplementary Material

Additional file 1: Table S1. Associated MIGS record. Click here for file
