# Supplementary material for: High-quality permanent draft genome sequence of Bradyrhizobium sp. Th.b2, a microsymbiont of Amphicarpaea bracteata collected in Johnson City, New York
Source: Stand Genomic Sci. 2015 May 16;10:24. doi: 10.1186/s40793-015-0008-y (PMC4511635; doi:10.1186/s40793-015-0008-y)
Supplement: Additional file 1: Table S1. — Associated MIGS record. [file s40793-015-0008-y-S1.pdf]

Additional file 1: Associated MIGS record

Table S1. Associated MIGS record for *Bradyhizobium* sp. Th.b2

MIGS-ID	field name	description
**MIGS-1**	Submit to INSDC/Trace archives	
**1.1**	PID	
**1.2**	Trace Archive	
**MIGS-2**	MIGS CHECK LIST TYPE	
**MIGS-3**	Project Name	GEBA - Root Nodulating Bacteria
**MIGS-4**	Geographic Location	Johnson City, New York
**4.1**	Latitude	42.107
**4.2**	Longitude	- 75.9691
**4.3**	Depth	
**4.4**	Altitude	
**MIGS-5**	Time of Sample collection	
**MIGS-6**	Habitat (EnvO)	Soil, root nodule, host
**6.1**	temperature	28
**6.2**	pH	
**6.3**	salinity	
**6.4**	chlorophyll	
**6.5**	conductivity	
**6.6**	light intensity	
**6.7**	dissolved organic carbon (DOC)	
**6.8**	current	
**6.9**	atmospheric data	
**6.10**	density	
**6.11**	alkalinity	
**6.12**	dissolved oxygen	
**6.13**	particulate organic carbon (POC)	
**6.14**	phosphate	
**6.15**	nitrate	
**6.16**	sulfates	
**6.17**	sulfides	
**6.18**	primary production	
**MIGS-7**	Subspecific genetic lineage	
**MIGS-9**	Number of replicons	
**MIGS-10**	Extrachromosomal elements	
**MIGS-11**	Estimated Size	
**MIGS-12**	Reference for biomaterial or Genome report	
**MIGS-13**	Source material identifiers	
**MIGS-14**	Known Pathogenicity	Non-pathogen
**MIGS-15**	Biotic Relationship	Symbiotic
**MIGS-16**	Specific Host	*Amphicarpaea bracteata*
**MIGS-17**	Host specificity or range (taxid)	
**MIGS-18**	Health status of Host	
**MIGS-19**	Trophic Level	
**MIGS-22**	Relationship to Oxygen	Aerobe
**MIGS-23**	Isolation and Growth conditions	TY media, 28°C, aerobe
**MIGS-27**	Nucleic acid preparation	CTAB
**MIGS-28**	Library construction	Illumina Standard PE libraries

**28.1**	Library size	3,052.2Mbp
**28.2**	Number of reads	20,348,156
**28.3**	vector	
**MIGS-29**	Sequencing method	Illumina HiSeq2000
**MIGS-30**	Assembly	
**30.1**	Assembly method	Velvet version 1.1.04; Allpaths-LG version r42328
**30.2**	estimated error rate	
**30.3**	method of calculation	
**MIGS-31**	Finishing strategy	
**31.1**	Status	Permanent Draft
**31.2**	coverage	120.4x
**31.3**	contigs	274
**MIGS-32**	Relevant SOPs	
**MIGS-33**	Relevant e-resources	
